# A Novel Ferrocene-Linked Thionine as a Dual Redox Mediator for the Electrochemical Detection of Dopamine and Hydrogen Peroxide

**DOI:** 10.3390/bios14090448

**Published:** 2024-09-19

**Authors:** Manikandan Palinci Nagarajan, Manikandan Ramalingam, Ilakeya Subbiah Arivuthilagam, Vishwa Paramaguru, Md. Mahbubur Rahman, Jongdeok Park, Francis Kwaku Asiam, Byungjik Lee, Kwang Pyo Kim, Jae-Joon Lee

**Affiliations:** 1 Research Center for Photoenergy Harvesting & Conversion Technology (phct), Department of Energy & Materials Engineering, Dongguk University, 26 Phil-dong, 3-ga, Jung-gu, Seoul 04620, Republic of Korea; 2 Department of Applied Chemistry, Kyung Hee University, 1732 Deokyoung-daero, Giheung-gu, Yongin-si 17104, Republic of Korea; 3 Department of Energy Materials Science and Engineering, Konkuk University, Chungju 27478, Republic of Korea; mahbub1982@kku.ac.kr

**Keywords:** redox mediator, electro-oxidation, limit of detection, ferrocene, thionine

## Abstract

We introduce a novel dual redox mediator synthesized by covalently linking ferrocene dicarboxylic acid (FcDA) and thionine (TH) onto a pre-treated glassy carbon electrode. This unique structure significantly enhances the electro-oxidation of dopamine (DA) and the reduction of hydrogen peroxide (H_2_O_2_), offering a sensitive detection method for both analytes. The electrode exhibits exceptional sensitivity, selectivity, and stability, demonstrating potential for practical applications in biosensing. It facilitates rapid electron transfer between the analyte and the electrode surface, detecting H_2_O_2_ concentrations ranging from 1.5 to 60 µM with a limit of detection (LoD) of 0.49 µM and DA concentrations from 0.3 to 230 µM with an LoD of 0.07 µM. The electrode’s performance was validated through real-sample analyses, yielding satisfactory results.

## 1. Introduction

The advancement of modern analytical techniques for human health management is driven by the need to develop sustainable, reliable, and user-friendly systems for monitoring analytes in various matrices, including environmental samples, food commodities, and biological fluids [1,2]. These techniques emphasize using environmentally friendly materials and reduced reagent quantities to minimize chemical waste production.

Hydrogen peroxide (H_2_O_2_) is not only industrially significant but also a well-known byproduct of numerous enzymatic reactions, making its accurate quantification critical in food industries, environmental monitoring, and clinical applications [3]. As a typical reactive oxygen species, H_2_O_2_ can induce oxidative stress damage to cells, including neurons. The brain, with its high oxygen consumption, elevated polyunsaturated fatty acid content, and relatively low antioxidant defenses, is particularly susceptible to oxidative stress. Evidence suggests that oxidative stress may play a role in neurodegenerative disorders characterized by the loss of dopamine (DA)-producing neurons [4]. Abnormal DA levels are linked to diseases such as Parkinson’s, Alzheimer’s, senile dementia, and schizophrenia [5,6]. Understanding the relevance or interplay between DA and oxidative stress levels correlated with H_2_O_2_ levels is of significant interest [7].

Direct electrochemical detection of DA [8,9,10,11,12,13,14,15,16] and H_2_O_2_ [17,18,19,20,21,22,23] is well-known but typically requires sophisticated electrode design for optimization, stability, and reproducibility over a wide concentration range. This is often achieved using nanomaterials, including conducting polymers [24,25], graphene [26,27,28,29], and redox mediators [30,31,32,33,34]. Early detection of DA and nitrophenol utilizing thionine (TH) as a redox mediator immobilized on glassy carbon (GC) electrodes has been reported [35,36,37,38]. Recent studies have highlighted the use of bi-mediators capable of detecting dual analytes. The complex form of nickel hexacyanoferrate and TH on GC electrodes were used for the oxidation of gallic acid and reduction of H_2_O_2_ [12,39], respectively, while the concept of bi- or dual-mediators for electrochemical sensors has been rarely explored.

**Scheme 1:**
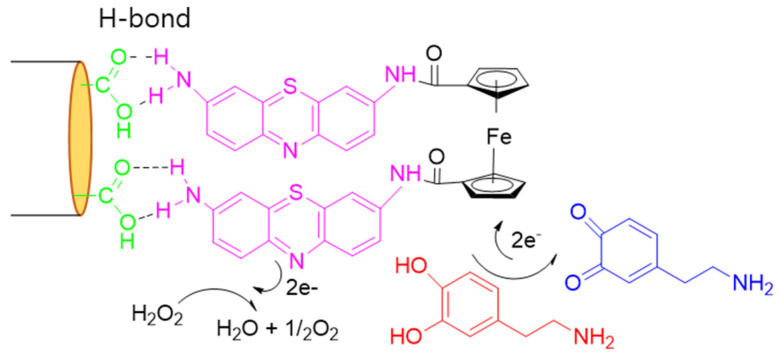
Electrochemical oxidation of DA and reduction of H_2_O_2_ on **GC/TH−FcDA**.

In this study, we developed a novel electrode with a complex dual redox mediator consisting of ferrocene dicarboxylic acid (FcDA) directly linked to a TH unit. The dual mediator was synthesized by covalently linking FcDA and TH, followed by immobilization onto a pre-treated GC surface via hydrogen bonding interactions, resulting in the formation of **GC/TH−FcDA** for electrochemical sensor applications, as shown in Scheme 1. We investigated the electrochemical behavior of **GC/TH−FcDA** for the electrocatalytic detection of both DA and H_2_O_2_ by each electrochemically active moiety of FcDA and TH over a wide potential range at a single electrode. **GC/TH−FcDA** exhibited stable and reproducible sensing capabilities for dual analytes and can be extended for simultaneous detection of multiple analytes through simple electrode design.

## 2. Materials and Methods

### 2.1. Reagents and Materials

All reagents used in this study were of analytical grade and purchased from Sigma-Aldrich (St.Louis, MO, USA) without further purification. Double-distilled water (18.2 MΩ∙cm), obtained from a Millipore Milli-Q bio cell A10 water purification system (Merck, Darmstadt, Germany), was employed throughout the experiments. TH, FcDA, N-ethyl-N’-(3-dimethylaminopropyl)-carbodiimide hydrochloride (EDC.HCl), hydroxybenzotriazole (HOBt), DA, ascorbic acid (AA), uric acid (UA), disodium hydrogen phosphate, sodium dihydrogen phosphate, and H_2_O_2_ were used as received. A phosphate-buffer solution (PBS, 0.1 M, pH 7) was prepared by mixing aqueous solutions of disodium hydrogen phosphate and sodium dihydrogen phosphate. All electrochemical measurements were conducted at ambient temperature. Before measurements, the electrolyte was purged with inert nitrogen gas for 5 min to eliminate active oxygen interference.

### 2.2. Instrumentation

Electrochemical experiments were conducted using a CHI-430A potentiostat (CH Instruments, Austin, TX, USA) with a standard three-electrode setup. The reference and auxiliary electrodes consisted of an Ag/AgCl electrode saturated with KCl and a platinum wire. The working electrodes were GC electrodes with a diameter of 3 mm, modified with TH-FcDA to obtain **GC/TH−FcDA**. Fourier-transform infrared (FT-IR) measurements were performed in AT-IR mode using a JASCO FT-IR-4600 instrument (Tokyo, Japan). The surface morphology of materials was characterized by scanning electron microscopy (SEM, JEOL JSM-7100, Tokyo, Japan).

### 2.3. Preparation of TH-FcDA

**Figure 1:**
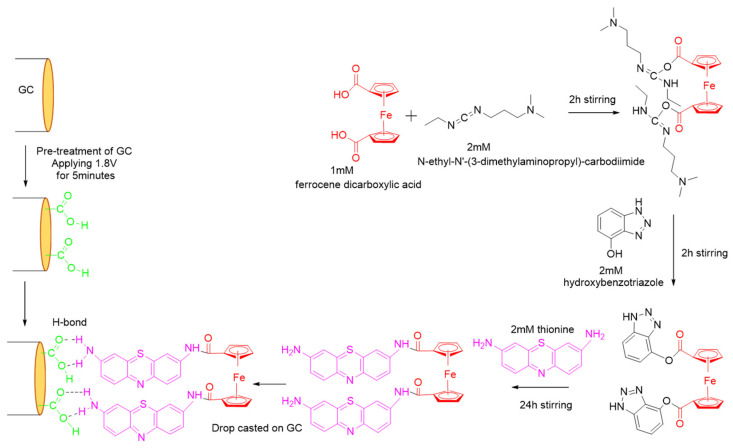
Schematic illustration of the preparation process of the thionine-linked ferrocene dicarboxylic acid-modified GC electrode.

TH was covalently linked to FcDA in a 2:1 molar ratio using EDC.HCl and HOBt as cross-linking reagents [12], as depicted in Figure 1. FcDA (1 mM) and 2 mmol of EDC.HCl were stirred for 2 h to activate the carboxylic acid (-COOH) groups of ferrocenes for o–acylurea formation. Subsequently, 2 mM of HOBt was added to the reaction mixture and stirred vigorously for another 2 h to facilitate ester formation. Then, 2 mmol of TH was introduced, and the reaction mixture was stirred vigorously to promote the reaction of the amine (-NH_2_) group in TH with the ester group, resulting in an amide bond formation. The covalently bonded TH-FcDA material was precipitated, washed with ethanol to remove unreacted precursors, and dried in a vacuum oven at 80 °C for 6 h and used for further electrochemical measurements.

### 2.4. Modification of GC with TH-FcDA

The GC was surface-cleaned and polished prior to modification. The GC’s surface was pre-treated using a chronoamperometric technique previously reported by our group [15,33,34]. The GC was immersed in 0.1 M PBS (pH 7) and subjected to a constant potential of +1.8 V for 300 s. The TH-FcDA solution (5 mg/mL in dimethyl sulfoxide, 3 µL) was then drop-casted onto the pre-treated GC surface and dried at ambient temperature. The modified electrode (**GC/TH−FcDA**) was subsequently washed with water to remove any unbonded material and dried under an inert atmosphere.

## 3. Results and Discussions

### 3.1. FT-IR and Morphology Studies

**Figure 2:**
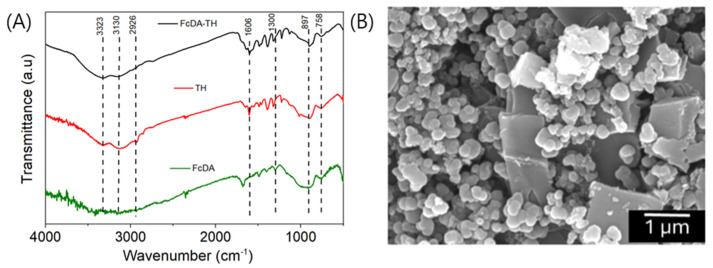
(**A**) FT-IR spectra of FcDA, TH, and TH-FcDA. (**B**) SEM images of TH-FcDA.

The chemical groups of FcDA, TH, and TH-FcDA were characterized via FT-IR, as shown in Figure 2A. The spectra revealed the presence of the -COOH group in FcDA, the NH_2_ group in TH, and the amide group in TH-FcDA. The IR bands at 758 and 897 cm^−1^ correspond to the aromatic C-H bending in all three materials. In FcDA, the bands at 1300 and 1672 cm^−1^ confirmed the presence of C-O and C=O stretching vibrations of the -COOH group, respectively [40]. In addition, the IR band at 2926 cm^−1^ corresponds to the C-H stretching in TH [41]. Furthermore, the peaks at 3130 and 3323 cm^−1^ verified the existence of -NH_2_ group in TH. Conversely, the C=O stretching vibration shifted to 1606 cm^−1^ with slight broadening owing to the at -CO-NH- bond formation in TH-FcDA. Therefore, the FT-IR analysis confirmed the formation of the TH-FcDA complex. The morphologies of these materials were analyzed using SEM measurements. The FcDA showed an aggregated and interconnected nanoparticle structure (Figure S1A). In addition, the TH revealed the aggregated nanonetwork-like morphology (Figure S1B). As shown in Figure 2B, the TH-FcDA exhibited a mixed structure with the combination of the interconnected and cluster type of nanoparticle structure and cubic type structure. These structural variations between the materials suggest the formation of TH-FcDA.

### 3.2. Electrochemical Measurements

**Figure 3:**
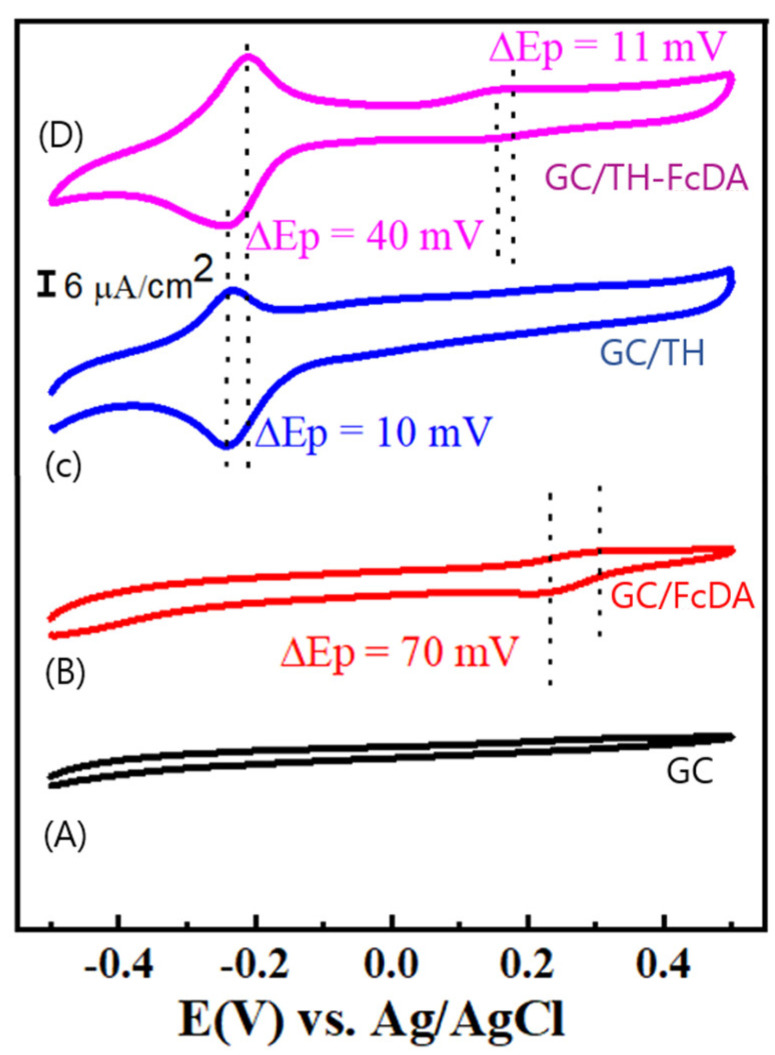
CV behavior of the **GC** (curve **A**), **GC/FcDA** (curve **B**), **GC/TH** (curve **C**), and **GC/TH−FcDA** (curve **D**) measured in 0.1 M PBS of pH 7.

The cyclic voltammetric (CV) behaviors of **GC**, **GC/FcDA**, **GC/TH**, and **GC/TH−FcDA** were investigated in a 0.1 M PBS at pH 7 with a scan rate of 50 mV/s, and the results are presented in Figure 3. The ferrocene oxidation and reduction peaks (E_pa_ and E_pc_, respectively) were observed at 0.28 and 0.21 V, respectively, with a ∆*E*_p_ of 70 mV for **GC/FcDA**. In comparison, the redox peaks for the formation of TH to leuco–TH were located with E_pa_ and E_pc_ of −0.21 and −0.18 V, respectively, with ∆*E*p of 30 mV for **GC/TH**. In the redox complex of the TH-FcDA-modified GC, the ferrocene redox peak shifted negatively, and the current also increased, indicating the TH-mediated redox reaction of FcDA. The TH redox peak current increased in the **GC/TH−FcDA** electrode compared to the **GC/TH** electrode (ΔE_p_ = 10 mV) with the ΔE_p_ of 40 mV. This can be attributed to the improved charge transfer properties of the linked moieties resulting from the hydrogen bonding interactions between the -NH_2_ groups of TH and –COOH functionalities of GC, as schematically presented in Figure 1.

**Figure 4:**
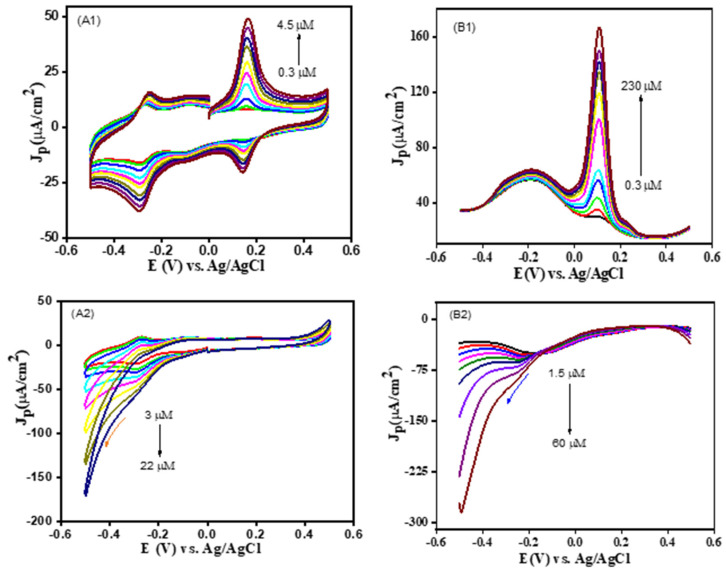
(**A**) CV and (**B**) DPV responses of **GC/TH−FcDA** in 0.1 M PBS (pH 7) at different concentrations of DA (**A1** and **B1**) and H_2_O_2_ (**A2** and **B2**).

Figure 4A illustrates the CV response of **GC/TH−FcDA** in 0.1 M PBS at pH 7, with DA concentrations varying from 0.3 to 4.5 mM and H_2_O_2_ concentrations from 3 to 22 µM. The results demonstrate a linear increase in the anodic peak current density (J_pa_) within the redox potential of FcDA (0.16 V) upon adding DA, with a linear regression equation of J_p_ (mA/cm^2^) = 9.40 [DA] (mM) + 7.01 and a correlation coefficient, R^2^ = 0.9983 (Figure S2A1). The reduction peak current of TH also increases as the concentration of DA increases in successive CV measurements. This is likely due to the polymerization of DA onto the electrode surface. Additionally, a linear increase in cathodic peak current density (J_pc_) was observed at −0.2 V with increasing H_2_O_2_ concentrations, corresponding to a linear regression equation of J_p_ (µA/cm^2^) = 2.07 [H_2_O_2_] (µM) + 14.08 (R^2^ = 0.9993) (Figure S2A2). These demonstrate the low LoDs of 2.77 and 1.37 µM for DA and H_2_O_2_, respectively. [12,38] These CV findings suggest that the oxidation of DA to dopamine-o-quinone occurs at FcDA moieties, while the reduction of H_2_O_2_ to H_2_O and ½O_2_ occurs at TH moieties (Scheme 1). This was further confirmed using density functional theory (DFT), as detailed in Section 3.5.

Moreover, the different pulse voltammetry (DPV) response of **GC/TH−FcDA** in 0.1 M PBS at pH 7 with DA concentrations ranging from 0.3 to 230 μM and H_2_O_2_ from 1.5 to 60 μM is shown in Figure 4B. The calibration curve for the electrochemical detection of DA and H_2_O_2_ using the **GC/TH−FcDA** demonstrated a good linear relationship between the peak currents and the concentrations, spanning 0.3 to 230 µM for DA and 1.5 to 60 µM for H_2_O_2_ (Figure S2B). The electrode exhibited a linear regression equation of J_pa_ (µA/cm^2^) = 0.59 [DA] (µM) + 29.84 with an R^2^ of 0.9997 and an estimated sensitivity of 0.59 µA/µM/cm^2^, indicating the high electrocatalytic activity towards the oxidation of DA at 0.16 V. The LoD was determined to be 0.07 µM, which is lower than previously reported values for similar electrodes (Table S1A) [12,13,14,15,16,17,18,19]. Furthermore, for H_2_O_2_ detection, the electrode demonstrated a sensitivity of 0.68 µA/µM/cm^2^ with a linear regression equation of J_pc_ (µA/cm^2^) = 0.68 [H_2_O_2_] + 57.23 with R^2^ = 0.9996 and an LoD (S/N = 3) of 0.49 µM, exceeding earlier findings (Table S1B) [12,21,22,23,24,25,26,27].

**Figure 5:**
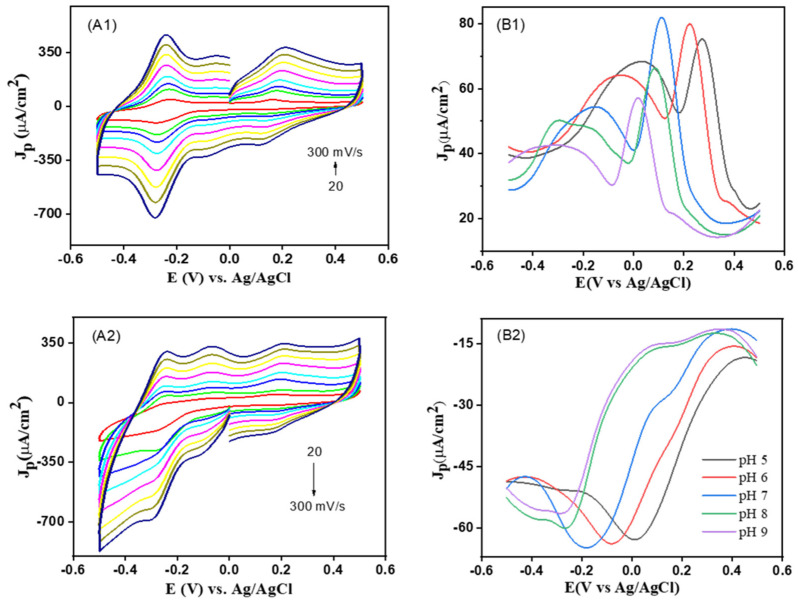
CV response of **GC/TH−FcDA** in 0.1 M PBS (pH 7) in the presence of (**A1**) 10 μM DA and (**A2**) 10 μM H_2_O_2_ at different scan rates (10–300 mV/s). DPV response of **GC/TH−FcDA** in 0.1 M PBS at different pHs (5–9) in the presence of (**B1**) 10 μM DA and (**B2**) 10 μM H_2_O_2._

Figure 5(A1,A2) presents the CV response of **GC/TH−FcDA** in 0.1 M PBS at pH 7, featuring both 10 µM of DA and H_2_O_2_, with scan rates ranging from 10 to 300 mV/s. The voltammograms reveal that both the J_pa_ and J_pc_, corresponding to the redox potential of FcDA moieties at 0.15 V, indicative of DA oxidation, increase with the square root of the scan rate. These observations suggest that the DA oxidation process is diffusion-controlled, consistent with findings reported in an earlier study [38]. Similarly, the J_pc_ for the reduction of H_2_O_2_ increased with the scan rate, which showed linear behavior between J_pc_ and the square root of the scan rate, indicating a diffusion-controlled reduction reaction of H_2_O_2_, aligning with previous reports [19].

DPV measurements were conducted on **GC/TH−FcDA** to evaluate the influence of supporting electrolyte pH on DA oxidation and H_2_O_2_ reduction, as depicted in Figure 5(B1,B2). The J_pa_ for DA oxidation increased gradually from pH 5 to 7, followed by a decline from pH 7 to 9 (Figure 5(B1)). The E_pa_ shifted negatively with increasing pH from 5 to 9, described by the linear regression equation E_pa_ = -0.065pH + 0.595 (R^2^ = 0.995) (Figure S3A). This result suggests that the stoichiometry for the DA oxidation reaction involved two electrons and two protons [42]. Again, the J_pc_ for H_2_O_2_ reduction increased with increasing pH from 5 to 7, then decreased from pH 7 to 9 (Figure 5(B2)). The E_pc_ also shifted negatively with rising pH from 5 to 9, quantified by the linear regression equation E_pc_ = -0.078pH + 0.078 (R^2^ = 0.993) (Figure S3B). The number of electrons and protons involved in the H_2_O_2_ reduction was determined to be two [19]. The J_pa_ for DA oxidation and J_pc_ for H_2_O_2_ reduction reached their maximum at pH 7 (0.1 M PBS), establishing pH 7 (0.1 M PBS) as the optimal supporting electrolyte pH for sensing DA and H_2_O_2_.

### 3.3. Interference Study

**Figure 6:**
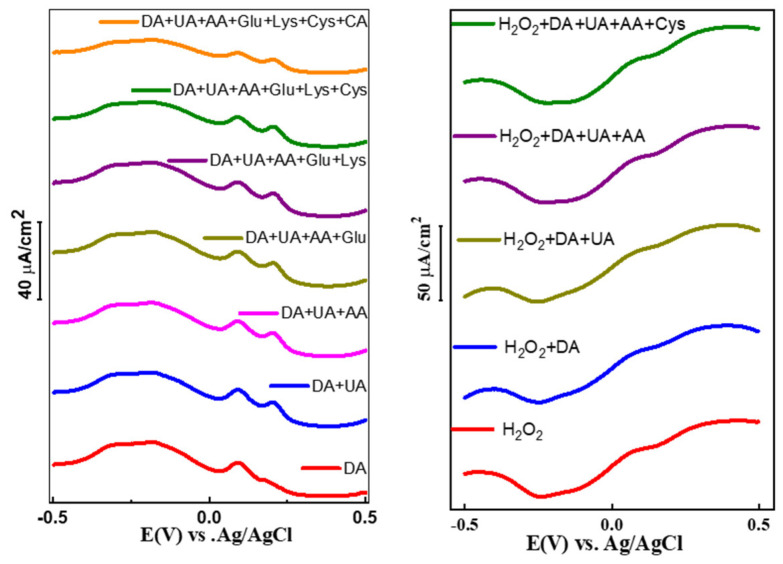
DPV response of 10 µM DA and H_2_O_2_ with 300 µM of different interference species in 0.1 M PBS pH 7.

The detection of DA (10 µM) and H_2_O_2_ (10 µM) in the presence of a 100-fold excess of various analytes, including UA, AA, glucose, lysine, cysteine, and catechol, was investigated, and the obtained DPV results are displayed in Figure 6. An additional oxidation peak at 0.21 V was noted, signifying the oxidation of UA. Importantly, this peak did not interfere with the DA oxidation, attributed to a potential difference of 0.05 V between the oxidation processes of DA and UA. These findings confirm that excess concentrations of other analytes did not affect the detection of DA, and none of the above analytes interferes with the detection of H_2_O_2_, demonstrating the good selectivity of the **GC/TH−FcDA** sensor.

### 3.4. Real-Sample Analysis, Reproducibility, and Stability

To examine the practical applicability of the proposed **GC/TH−FcDA sensor**, a real-sample analysis was conducted. Human urine samples were pre-treated and stored at 4 °C prior to analysis [38]. H_2_O_2_ was detected in commercial milk samples. Both DA and H_2_O_2_ detections were performed using the standard addition method [39]. Five repetitive experiments were conducted for each sample, and recovery measurements were also performed using the standard addition method. The recovery results for DA ranged from 99.8% to 100.08%, while for H_2_O_2_, recoveries ranged from 99.2% to 101.0%, as delineated in Table 1. These results substantiate the excellent recovery performance and practical applicability of the developed electrode material.

As depicted in Figure S4, the **GC/TH−FcDA** demonstrated excellent reproducibility with a relative standard deviation (RSD%) of 0.35% and 0.07%, estimated from 30 consecutive CV scans performed in 10 µM H_2_O_2_ and DA 0.1 M PBS pH 7, respectively. For a long-term stability study, the **GC/TH−FcDA** sensor was stored in an airtight container when not in use for 20 days and retained 90% of its initial response. These findings confirm the stability of the electrode and its capacity to resist interference from repeated use cycles.

### 3.5. Density Functional Theory (DFT) Studies

**Figure 7:**
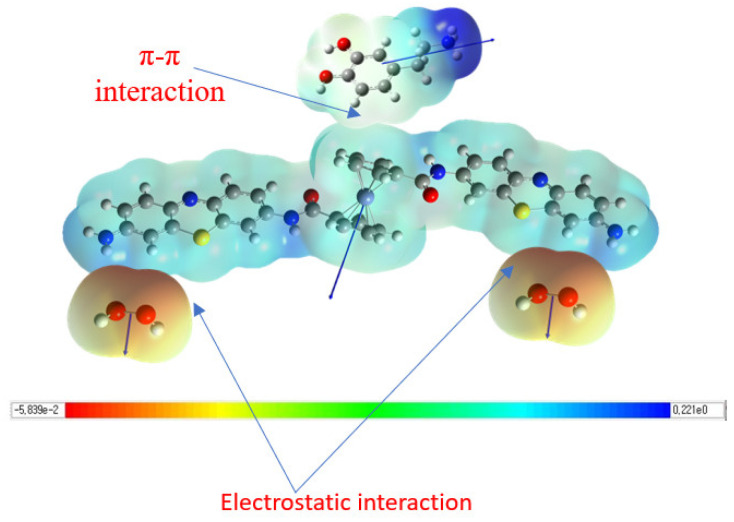
Electrostatic potential (ESP) map for TH-FcDA with DA and H_2_O_2_.

The geometries and corresponding properties of the computed structures were analyzed using the B3LYP/6-31G (d,p) [43] level of theory with the GAUSSIAN 09 software and visualized with GaussView (5). No imaginary modes were observed in the frequency analysis, confirming that all structures are stationary points. The corresponding geometric coordinates are tabulated in Table S2 [44]. Figure 7 presents the electrostatic potential (ESP) map of TH-FcDA and its interactions with DA and H_2_O_2_. The DA molecule interacts with TH-FcDA through a potential π-π interaction site on both DA and FcDA molecules. The ESP map of the two materials reveals that the net positive density of TH-FcDA is localized on one side of the FcDA, while the net negative density on DA is oriented towards the aromatic ring, suggesting possible repulsion of DA on the TH side due to the positive charge density at the edges. Additionally, H_2_O_2_ demonstrates an electrostatic interaction with TH, primarily utilizing the oxygen groups at the positive end of TH.

## 4. Conclusions

This research successfully developed a highly stable dual redox mediator by covalently linking FcDA and TH onto a GC electrode. The electrochemical investigations elucidated the roles of FcDA and TH moieties in facilitating the oxidation of DA and reduction of H_2_O_2_, respectively. Carboxylation of the GC surface enhanced the immobilization of the redox mediator, resulting in high stability and a reproducible sensing response under neutral pH conditions. The electrode exhibited a notable sensing performance for DA, with a detection range from 0.3 to 230 µM, a sensitivity of 0.59 µA/µM/cm^2^, and a LoD of 0.07 µM (S/N = 3). Additionally, the electrode enabled effective sensing of H_2_O_2_, displaying in a range from 1.5 to 60 µM, with a sensitivity of 0.68 µA/µM/cm^2^ and an LoD of 0.49 µM (S/N = 3). Notably, the sensor was validated by detecting DA and H_2_O_2_ in diluted urine and milk samples, demonstrating its potential for practical applications in biosensing.

## Figures and Tables

**Table 1:** Estimation of DA in diluted human urine samples. Estimation of H_2_O_2_ in milk samples.

Sample No	Spiked (µM)	Found (µM) *	Recovery (%)	RSD (%)
1	10	9.98 ± 0.02	99.8	0.2
2	50	50.04 ± 0.04	100.08	0.08
3	100	99.89 ± 0.11	99.89	0.11
**Sample No**	**Spiked (µM)**	Found (µM) *	**Recovery (%)**	**RSD (%)**
1	5	4.96 ± 0.04	99.2	0.8
2	10	10.10 ± 0.10	101	0.99
3	20	20.09 ± 0.09	100.45	0.44

* Average three-replicate measurements.

## Data Availability

The original contributions presented in the study are included in the article; further inquiries can be directed to the corresponding authors.

## Supplementary Materials

The following supporting information can be downloaded at: https://www.mdpi.com/article/10.3390/bios14090448/s1. Figure S1. SEM images of (A) FcDA, and (B) TH. Figure S2. Linear plot for different concentrations of DA (A1, B1) and H_2_O_2_ (A2, B2) measured from Figure 4. Figure S3. Linear plot for different pH in the detection of DA (A) and H_2_O_2_ (B) measured from Figure 5(B1,B2). Figure S4. Reproducibility and Stability of **GC/TH−FcDA** electrode in the presence of H_2_O_2_ (A1,A2) and DA (B1,B2). Table S1. A. Comparison of analytical parameters for DA detection at **GC/TH−FcDA**. B. Comparison of analytical parameters for H_2_O_2_ detection at **GC/TH−FcDA**. Table S2. Cartesian coordinates of the molecules.
