# Supplementary material for: A Novel Ferrocene-Linked Thionine as a Dual Redox Mediator for the Electrochemical Detection of Dopamine and Hydrogen Peroxide
Source: Biosensors (Basel). 2024 Sep 19;14(9):448. doi: 10.3390/bios14090448 (PMC11429643; doi:10.3390/bios14090448)
Supplement: Supplementary file 1 [file biosensors-14-00448-s001.zip › biosensors-3025777-supplementary.pdf]

biosensors

Supporting information

A Novel Ferrocene-linked Thionine as a Dual Redox Mediator for the Electrochemical Detection of Dopamine and Hydrogen Peroxide

A Novel Ferrocene-Linked Thionine as a Dual Redox Mediator for the Electrochemical Detection of Dopamine and Hydrogen Peroxide

Manikandan Palinci Nagarajan [1,2], Manikandan Ramalingam [1], Ilakeya Subbiah Arivuthilagam [1], Vishwa Paramaguru [1], Md. Mahbubur Rahman [3], Jongdeok Park [1], Francis Kwaku Asiam [1], Byungjik Lee [1], Kwang Pyo Kim [2,*] and Jae-Joon Lee [1,*]

[1] Research Center for Photoenergy Harvesting & Conversion Technology (phct), Department of Energy & Materials Engineering, Dongguk University, 26 Phil-dong, 3-ga, Jung-gu, Seoul 100-715, Republic of Korea
[2] Department of Applied Chemistry, Kyung Hee University, 1732 Deokyoung-daero, Giheung-gu, Yongin-Si 17104, Republic of Korea
[3] Department of Energy Materials Science and Engineering, Konkuk University, Chungju 27478, Republic of Korea; mahbub1982@kku.ac.kr
* Correspondence: kimkp@khu.ac.kr (K.P.K.); jjlee@dongguk.edu (J.-J.L.)

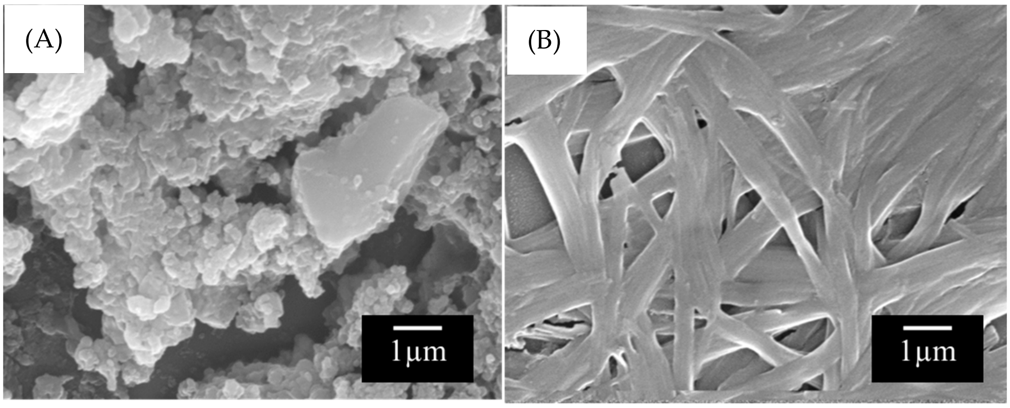

Figure S1. SEM images of (A) FcDA, and (B) TH.

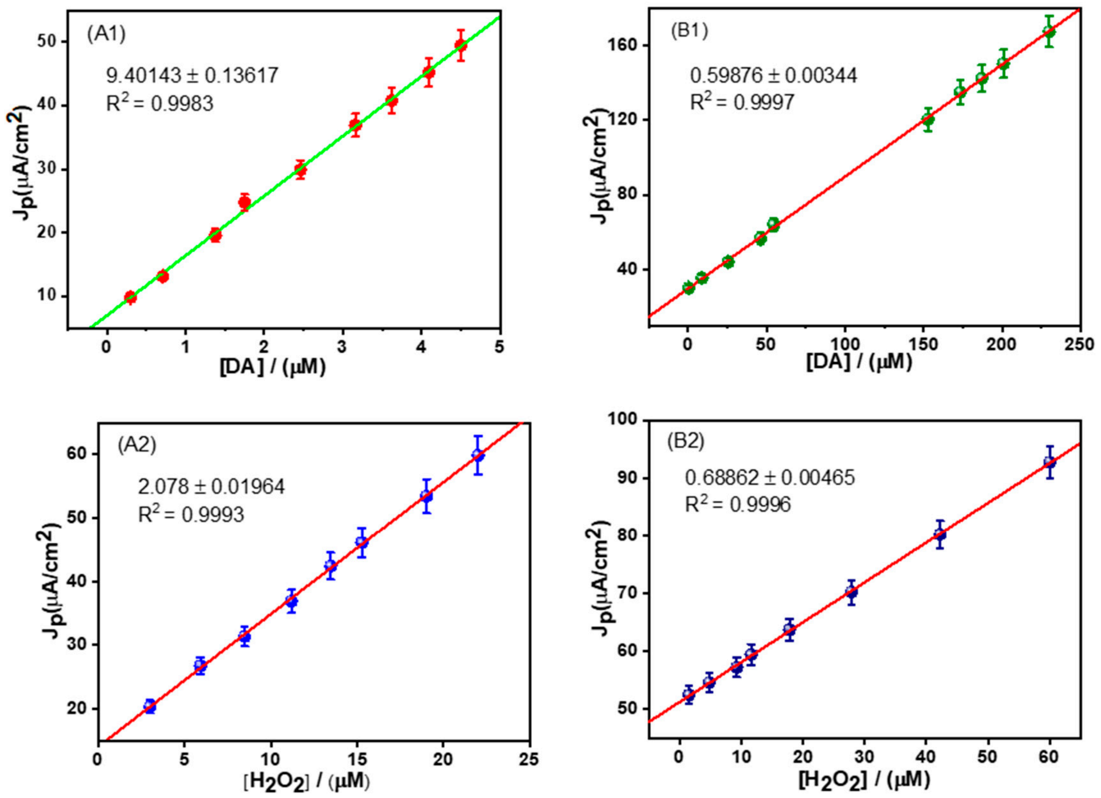

Figure S2. Linear plot for different concentrations of DA (A1, B1) and H_2O_2 (A2, B2) measured from Figure 4.

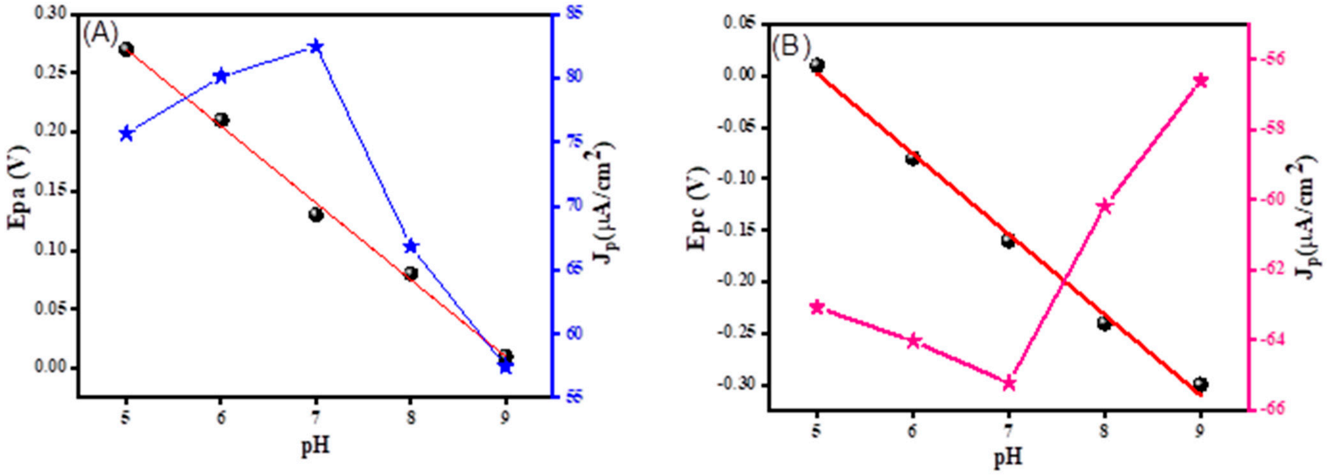

Figure S3. Linear plot for different pH in the detection of DA (A) and H_2O_2 (B) measured from Figure 5(B1,B2).

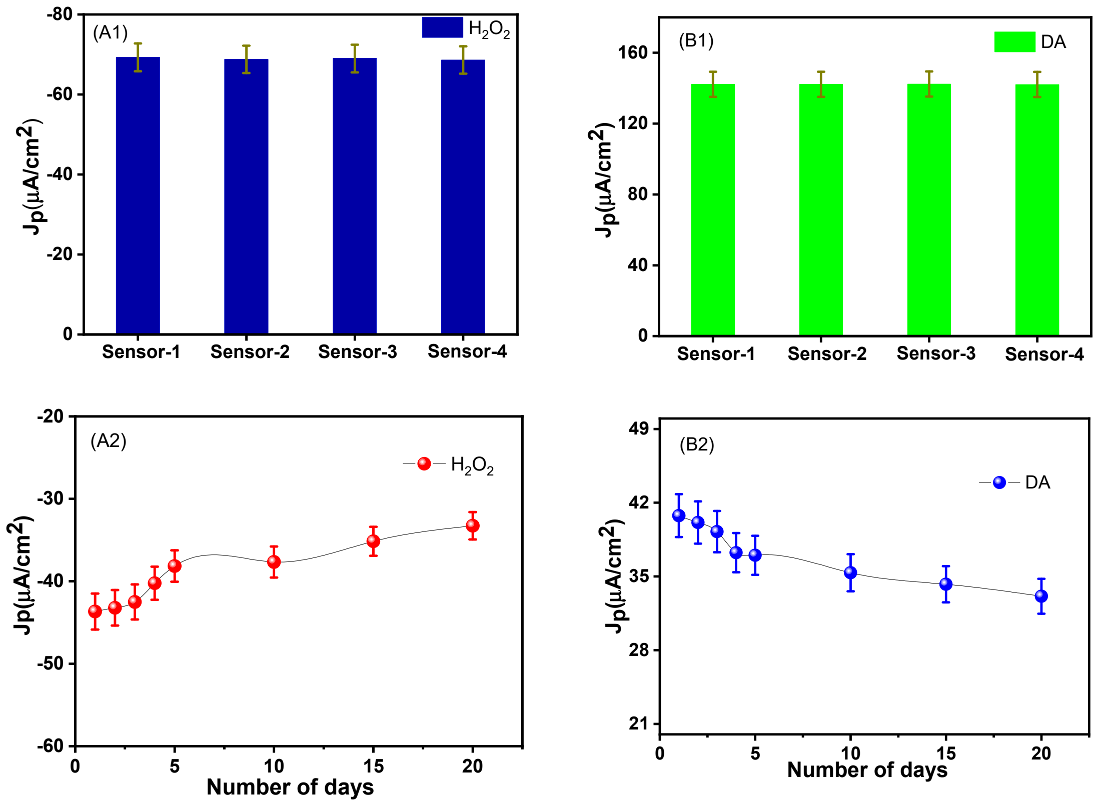

Figure S4. Reproducibility and Stability of **GC/TH–FcDA** electrode in the presence of H_2O_2 (A1,A2) and DA (B1,B2)

Table S1. A. Comparison of analytical parameters for DA detection at **GC/TH–FcDA**. B. Comparison of analytical parameters for H_2O_2 detection at **GC/TH–FcDA**.

**A**						
**Modified electrode Material**	**Technique**	**Linear range (µM)**	**LOD (µM)**	**Sensitivity (µM/µA/cm²)**	**Electrolyte**	**Ref**
Fe_3O_4@APTES/SPCE	DPV	1-200	0.034	-	pH 6.5	12
L_j-$LaMnO_3$/GCE	CA	1-600	0.032	1	0.1 M KOH	13
Cu/Cu_xO NPs/PGE	DPV	0.3-53	1.07	0.51	pH 5.8	14
GO/WO_3/GCE	CA	0.3 -1245	0.306	0.392	pH 7	15
Graphene-GQDs/GCE	DPV	0.1-100	0.03	14.25	pH 6	16

La-doped MTO/GCE	CA	5 - 50	1.32	-	pH 7	17
MoS_2/CC	CV	250 – 4000	0.3	0.77	pH 7.4	18
GCE/Poly(Co-TABAPc)	CV	0.1–4	30	0.016	pH 7	20
	AMP	0.1–1	20	0.024		
GCE/TH/Fc	CA	0.569 - 515	0.19	0.142	pH 7	19
**GC/TH-FcDA**	DPV	0.3 - 230	0.07	0.59	pH 7	This work
			**B**			
**Modified electrode Material**	**Technique**	**Linear range (μM)**	**LOD (μM)**	**Sensitivity (μM/μA/cm^2)**	**Electrolyte**	**Ref**
γ-Fe_2O_3/Fe_3O_4/GCE	CA	0.2 – 8000	0.05	-	pH 7.3	12
NiCo-DH/AuPt@CC	CA	10 – 670	0.145	-	pH 7.4	21
CuO/g-C_3N_4/GCE	DPV	0.5 - 50	0.31	3.327	pH 7	22
Bi_2Te_3/GCE	CA	-	0.016	4900	0.1 M K_2SO_4	23
AuNPs-PB-GO/GCE	CA	3.8-5400	1.3	87.6	0.5 M KCl	24
Cu-TCPP MOF/Cu5.4O/GCE	SWV	0.1 – 590	0.13	-	pH 7.4	25
AuNPs-Psi/GCE	LSV	2000 – 13810	14.84	10.65	pH 7	26
	SWV	500 - 6910	15.16	10.41		
GCE/TH/Fc	CA	0.569 - 785	0.19	0.128	pH 7	19
**GC/TH−FcDA**	DPV	1.5 - 60	0.49	0.68	pH 7	This-work

Table S2. Cartesian coordinates of the molecules.

Dopamine (E= −517.05647668 Hartrees)
N,0,0.9413134941,2.3284959067,−0.8435697701
C,0,−0.0547587588,1.2525397238,−0.861837543
C,0,−1.1294896503,1.4998214291,0.2098012972
C,0,−2.1574467495,0.3917579538,0.2790414341
C,0,−1.8836330921,−0.780616316,1.0002802441

C,0,−2.799453457,−1.8265127495,1.0436734207
O,0,−2.5158297444,−2.9558063795,1.7538616575
C,0,−4.0190146371,−1.7089483704,0.3546625517
O,0,−4.857674478,−2.7983777721,0.463600185
C,0,−4.3050257643,−0.554112137,−0.3620500044
C,0,−3.3761913022,0.4911429425,−0.3989805841
H,0,1.6826268995,2.1244293094,−1.5096709094
H,0,0.5123621164,3.1964035364,−1.1594837038
H,0,0.460055632,0.3112253244,−0.6361312962
H,0,−0.5536254952,1.1084420062,−1.8367272536
H,0,−1.6307240444,2.4529381815,−0.0043531492
H,0,−0.6241094287,1.6209450904,1.1741664928
H,0,−0.9515382522,−0.8935364848,1.5462221704
H,0,−3.2736863322,−3.5528603927,1.6629041614
H,0,−5.6719212262,−2.6324565619,−0.0269766611
H,0,−5.2530363282,−0.4664419647,−0.888943536
H,0,−3.6110994011,1.3916277244,−0.9592892042
Hydrogen peroxide (E= −151.54319300 Hartrees)
O,0,−0.718451786,−0.1175410519,−0.0544959339
O,0,0.7182804842,0.1175465486,−0.0542849118
H,0,−1.0140016216,0.6668525257,0.4349664917
H,0,1.0137211477,−0.6670772701,0.4348764909
Thionine (E= −1025.56236704 Hartrees)
C,0,0.0381078325,0.344566668,−1.3633644918
C,0,−0.1545770431,−1.0569996177,−1.0835247307
S,0,−0.2231588336,−1.6353411898,0.5645104461
C,0,−0.0143285269,−0.1806195548,1.5107541846
C,0,0.1586096915,1.0975334767,0.8655851986
N,0,0.178586126,1.3090649538,−0.4518656246
C,0,0.0831676176,0.7396547649,−2.7400225561
C,0,−0.0496629514,−0.1646928556,−3.751231818
C,0,−0.2404597273,−1.5534767734,−3.4559520313
C,0,−0.2896835614,−1.9770456946,−2.1086910094
C,0,−0.0194031986,−0.288208217,2.8906467054
C,0,0.1461305884,0.8620765303,3.6946302061
C,0,0.3190994831,2.1394597805,3.06957974
C,0,0.3237800947,2.2430728279,1.7104347248
N,0,0.1429533859,0.7700568602,5.0384267194
N,0,−0.3706867822,−2.4393658184,−4.4621867459
H,0,0.2284051355,1.7955092074,−2.9396659459
H,0,−0.0125816195,0.1555062848,−4.7876784186
H,0,−0.4344022385,−3.0303325805,−1.8867620159
H,0,−0.1504039524,−1.2557852176,3.3662965666
H,0,0.4458463527,3.0199013218,3.6915781229
H,0,0.453062594,3.1991957775,1.2155660926
H,0,0.0234076206,−0.1146531029,5.5072417693
H,0,0.2606024526,1.5871461158,5.6174267188
H,0,−0.5060708684,−3.4229560468,−4.2860847973
H,0,−0.3364396704,−2.1432678924,−5.4254469856
Ferrocenedicarboxylic acid (E= −2027.87503716 Hartrees)
C,0,−0.757555,0.630403,1.497326
C,0,−0.033773,−0.459607,2.057883

H,0,0.843852,−0.381826,2.685448
C,0,−0.632822,−1.674342,1.605792
H,0,−0.292393,−2.673164,1.842158
C,0,−1.815144,0.084613,0.696085
C,0,−1.725743,−1.345073,0.758913
H,0,−2.383877,−2.029406,0.243623
C,0,0.757542,0.630635,−1.497172
C,0,0.033724,−0.459240,−2.057906
C,0,0.632747,−1.674058,−1.606061
H,0,0.292298,−2.672831,−1.842620
C,0,1.815127,0.084675,−0.696073
C,0,1.725692,−1.344980,−0.759141
H,0,2.383825,−2.029427,−0.243996
Fe,0,0.000026,−0.541283,−0.000001
H,0,−0.843876,−0.381290,−2.685487
H,0,−0.551919,1.682104,1.633452
C,0,2.797248,0.811388,0.124942
C,0,−2.797228,0.811438,−0.124873
O,0,−3.631750,0.292394,−0.839903
O,0,−2.679040,2.159417,−0.002786
O,0,3.631790,0.292262,0.839886
O,0,2.679066,2.159393,0.003055
H,0,3.362902,2.536942,0.580977
H,0,−3.362888,2.537056,−0.580630
H,0,0.551966,1.682360,−1.633192
Ferrocenedicarboxylic acid linked with thionine (E= −3926.09245638 Hartrees)
C,0,0.883195,0.091454,2.304717
C,0,−0.357083,0.784068,2.314382
H,0,−7.263936,2.875513,−0.790350
C,0,−0.276016,1.865274,1.385657
H,0,−1.075572,2.544881,1.124653
C,0,1.754102,0.758415,1.378408
C,0,1.020378,1.856500,0.803484
H,0,1.361715,2.537545,0.034587
C,0,−1.198322,−1.475832,0.009209
C,0,0.138723,−1.858979,−0.289110
C,0,0.650897,−0.955719,−1.268821
H,0,1.646139,−0.983488,−1.690557
C,0,−1.517240,−0.322014,−0.791080
C,0,−0.355425,−0.001302,−1.572893
H,0,−0.290239,0.819172,−2.272932
Fe,0,0.077375,0.074059,0.425605
H,0,0.682383,−2.673410,0.168821
H,0,−1.219631,0.525446,2.912902
C,0,−2.767802,0.467513,−0.854106
C,0,3.143042,0.314419,1.128268
O,0,3.534073,−0.822693,1.342512
O,0,−2.802876,1.645346,−1.170253
H,0,−1.836373,−1.949850,0.744105
C,0,−12.689465,−0.939641,0.120163
C,0,−11.439128,−1.601469,0.140147
C,0,−10.269645,−0.885426,−0.034739

C,0,−10.297936,0.549193,−0.241326
C,0,−11.586778,1.187334,−0.253262
C,0,−12.736668,0.482080,−0.080984
C,0,−7.973545,0.881500,−0.442309
C,0,−7.571693,−0.484106,−0.266646
C,0,−6.228070,−0.834876,−0.306560
H,0,−5.935774,−1.873273,−0.174561
C,0,−5.240432,0.143164,−0.525950
C,0,−5.620381,1.505977,−0.703125
C,0,−6.946993,1.846853,−0.658065
H,0,−11.409534,−2.676303,0.292853
H,0,−11.595790,2.260461,−0.408197
H,0,−13.700118,0.981782,−0.094302
H,0,−4.854535,2.245781,−0.881407
N,0,−9.241279,1.329224,−0.423499
S,0,−8.751993,−1.744492,−0.000707
N,0,−3.922751,−0.275892,−0.550831
N,0,−13.832435,−1.624060,0.286747
H,0,−13.836171,−2.623312,0.427963
H,0,−14.727843,−1.158774,0.271357
H,0,−3.772762,−1.268875,−0.428111
C,0,12.262593,−1.609212,−0.508802
C,0,10.933977,−1.931819,−0.148115
C,0,9.953158,−0.956696,−0.156104
C,0,10.264557,0.408536,−0.531964
C,0,11.625901,0.698893,−0.893566
C,0,12.588641,−0.261332,−0.885271
C,0,8.102665,1.303437,−0.259383
C,0,7.445822,0.098110,0.154136
C,0,6.087610,0.074479,0.461080
H,0,5.595886,−0.833646,0.782315
C,0,5.340873,1.260032,0.367311
C,0,5.976787,2.469085,−0.043954
H,0,10.693026,−2.952032,0.135752
H,0,11.846860,1.723111,−1.172529
H,0,13.611099,−0.022892,−1.160735
H,0,5.392501,3.381867,−0.113893
H,0,7.810881,3.396047,−0.658161
N,0,9.405560,1.417845,−0.571481
S,0,8.329570,−1.401456,0.295263
N,0,3.990734,1.329557,0.658238
N,0,13.224413,−2.546487,−0.504483
H,0,13.032134,−3.501786,−0.242306
H,0,14.174756,−2.321868,−0.758844
H,0,3.580524,2.252801,0.610606
H,0,1.154836,−0.774187,2.891436
C,0,7.310250,2.486502,−0.345824
